# Challenges in Diagnosing Varicella-Zoster Virus Encephalitis in an Immunocompromised Patient

**DOI:** 10.7759/cureus.65226

**Published:** 2024-07-23

**Authors:** Andreana Chen, Prinka Perswani, Raj Kumari, Siddiqi M Haque, Min Zhang

**Affiliations:** 1 Internal Medicine, California University of Science and Medicine, Colton, USA; 2 Internal Medicine, University of California, Riverside, Riverside, USA; 3 Internal Medicine, St. Bernardine Medical Center, San Bernardino, USA

**Keywords:** end-stage renal disease (esrd), immunocompromised patient, clinical case report, varicella zoster virus encephalitis, varicella-zoster virus

## Abstract

Varicella-zoster virus (VZV) is a virus of the alphaherpesvirus family that is one of the common causes of infectious encephalitis worldwide, especially among those who are immunocompromised. In this case report, we discuss a case of a 55-year-old female with end-stage renal disease presenting with altered mental status and weakness. She was recently diagnosed with herpes zoster on oral acyclovir and multiple scattered dermatomal rashes on presentation. Cerebral spinal fluid analysis showed neutrophilic pleocytosis, high glucose and protein, and anti-VZV Immunoglobulin G (IgG) antibodies. She was started on treatment early with acyclovir and demonstrated good clinical improvement afterward two weeks. This case highlights to importance of performing lumbar puncture and looking for anti-VZV antibodies to rule out encephalitis in patients with altered mental status and starting acyclovir treatment early.

## Introduction

Varicella-zoster virus (VZV) is a double-stranded deoxyribonucleic acid (DNA) virus, part of the alphaherpesvirus family, that can present as two clinically distinct diseases: varicella (chickenpox) and zoster (shingles) [1]. Infection usually occurs in childhood, often in settings where vaccinations are limited, and develops into varicella. Afterward, the virus becomes latent in nerve ganglia and may resurface decades later as zoster. Zoster is commonly characterized by one or multiple painful, dermatomal vesicular rashes at multiple stages of healing. VZV reactivation most often occurs in elderly and immunocompromised patients, although it can happen at any age, with or without any triggers [2]. Complications of VZV reactivation include different neurological and ocular diseases, including giant cell Arteritis, encephalitis and meningitis, zoster sine herpete, and progressive outer retinal necrosis [2,3]. 

We present a case of a patient with VZV encephalitis caused by a combination of the patient having active virus reactivation in the form of shingles, in addition to being immunocompromised due to end-stage renal disease (ESRD). ESRD is a terminal complication of chronic kidney disease (CKD) characterized by renal failure, which is typically treated with long-term dialysis, and possibly a kidney transplant. Patients with ESRD are susceptible to high levels of uremia due to impaired renal clearance, and elevated serum urea is associated with suppression of the immune system [4]. According to the World Health Organization, encephalitis occurs in one out of every 33,000-50,000 cases of VZV [5]. It also carries a less favorable prognosis compared to the other extracutaneous complications of VZV. This case report shows how prompt recognition and treatment of this type of infection can decrease mortality and progression of the infection in a high-risk, immunocompromised patient.

## Case presentation

A 55-year-old female with a history of chicken pox in childhood and ESRD on peritoneal dialysis was admitted for a three-day history of altered mental status and weakness. This was the patient’s second healthcare encounter in the last one week. She was recently diagnosed with herpes zoster and was started on a 10-day course of oral acyclovir 800 mg. Her family reported that at her baseline, she was independent with her activities of daily living. In the last three days, she was reported to be thinking slower, and she has been in and out of consciousness. Eventually, she became unarousable, which prompted her presentation to the ER.

On admission, her temperature was 37.5 °C, white blood cell count was 7700 cells/uL (<5 cells/uL), sodium was 123 mEq/L (135-145 mEq/L), blood urea nitrogen (BUN) was 41 mg/dL (6 to 24 mg/dL), and creatinine was 13.48 mg/dL (0.6 to 1.1 mg/dL). Multiple scattered dermatomal rashes were observed on her right upper chest in a C4-C5 distribution and on her left lower back on the physical exam. On the neurological exam, she was drowsy and not oriented to age, month, or location but was able to follow simple commands. Cranial nerves II-VIII were intact. Muscle strength was 4/5 on all extremities. No focal neurological deficits were observed. The electroencephalogram (EEG) performed did not indicate any seizures but was positive for moderate generalized cerebral dysfunction of nonspecific etiology. Computed tomography (CT) of the head without contrast was not significant for any acute intracranial abnormalities. She was alert and oriented to person, place, and time the next day, although it unfortunately was short-lived, and her mental status declined on day 3 after admission. Throughout her stay, her mental status would wax and wane.

For her weakness, magnetic resonance imaging (MRI) of the brain, cervical, thoracic, and lumbar spine without contrast performed were unremarkable, which ruled down myelopathies. Based on the recent diagnosis of shingles, history of immunocompromise, and altered mental status, a lumbar puncture was performed. Cerebrospinal fluid (CSF) analysis revealed elevated white blood cells, with 79% neutrophils, 17% lymphocytes, and 4% monocytes, and elevated glucose and protein. CSF had high titers for Immunoglobulin G (IgG) for West Nile virus and VZV, whereas immunoglobulin M (IgM) VZV was low (Table 1). CSF culture was negative for growth. Encephalitis/meningitis polymerase chain reaction (PCR) panel of CSF came back negative for bacterial, viral, and fungal causes, including for VZV. PCR of blood drawn on day 9 was negative for VZV. Infectious disease, neurology, and hospital medicine teams all evaluated the patient and agreed with a diagnosis of VZV encephalitis in the setting of recent shingles, PCR of CSF, and patient presentation. The patient was administered a two-week course of acyclovir with improvement of her presenting symptoms prior to discharge.

**Table 1:** Cerebrospinal fluid analysis IgG: immunoglobulin G, IgM: immunoglobulin M, ISR: immune status ratio, IV: index value, VZV: varicella zoster virus

Test	Patient values	Reference ranges
White blood cells	51 cells/uL [H]	<5 cells/uL
Neutrophils	79% [H]	50-70%
Lymphocytes	17%	18-42%
Monocytes	4%	3.5-9.0%
Glucose	75 mg/dL [H]	40-70 mg/dL
Protein	121 mg/dL [H]	15-45 mg/dL
West Nile virus IgG	3.88 IV [H]	<=1.29 IV: negative 1.30 - 1.49 IV: equivocal >=1.50 IV: positive
VZV IgG	740.7 IV [H]	<=139.4 IV: negative 135.0 - 164.9 IV: equivocal >=165.0 IV: positive
VZV IgM	0.31 ISR	<=0.90 ISR: negative 0.91 - 1.09 ISR: equivocal >=1.10 ISR: positive

## Discussion

VZV encephalitis is a relatively rare complication that can start with milder symptoms, such as headache, fever, and neck stiffness [1]. The diagnosis is typically confirmed using PCR of the CSF [1]. Early treatment is highly recommended, which is acyclovir 10-15 mg/kg every eight hours for 14 days, adjusting for renal dosing as needed [6]. In a recent retrospective single-center case series study studying 74 patients, most patients recover without complications, especially when treated early and among younger patients [7].

Some studies argue that most cases of VZV encephalitis are secondary to VZV vasculopathies, which can cause neurological symptoms like headache and altered mental status like encephalitis but carries an additional risk for stroke [8-9]. In many cases, vasculopathy is visible on MRI brain imaging, and other diagnostic features, such as a recent shingles rash, increased cells in CSF, and VZV DNA detected on PCR, are sometimes not present in up to a third of diagnosed patients [8].

Some of the criteria used to diagnose VZV encephalitis include an onset of rash closely associated with the onset of neurological symptoms, positive VZV on PCR testing, and intrathecal synthesis of VZV antibodies [6]. Typically, increased IgM indicates recent active infection and increased IgG indicates past infection. Our patient matches two out of the three criteria mentioned: she had negative PCR for VZV DNA in both CSF and blood. However, according to current literature, viral DNA often disappears as early as one to three weeks after the onset of neurological symptoms [3,10]. Overall, the most sensitive test is anti-VZV IgG antibodies in CSF [3,10].

The etiology of the patient’s mental status was difficult to determine initially. At presentation, the differential for her altered mental status was multifactorial. Etiologies considered include hyponatremia, uremia given her ESRD, and VZV encephalitis given the presence of dermatomal rashes. Her hyponatremia resolved on day 3, but her mental status had not improved then. Her renal function was stable during her stay, so the cyclic nature of improving and declining mental status observed was also not suggestive of uremia as a cause. Therefore, VZV was our top differential.

The patient’s CSF had neutrophilic pleocytosis, high glucose, and protein, which is mostly suggestive of a viral cause, although typically there would be lymphocytic pleocytosis. However, PCR tests of both CSF and blood could not detect any infectious agents. Given her CSF profile and her existing dermatomal rash, she was diagnosed with VZV encephalitis. Her significant improvement in mentation and resolution of her rash after acyclovir treatment also supports this diagnosis.

ESRD is known to impair the immune system, which makes affected patients more vulnerable to infections and subsequent infection-related deaths [11]. A systematic review of varicella cases among ESRD adults found that this population was at a higher risk of developing serious complications, such as pneumonia, hepatitis, and encephalitis, like our patient, after contracting varicella [12]. The review also notes that in these studies, between 42% and 100% of ESRD patients who develop varicella do not have previous immunization against the virus [12]. Current Centers for Disease Control (CDC) and Kidney Disease: Improving Global Outcomes (KDIGO) guidelines recommend patients with CKD to receive the herpes zoster vaccine (Shingrix) [13,14]. With these in mind, it is important to advocate for and encourage ESRD patients to get vaccinated to reduce morbidity and mortality in this population from varicella.

## Conclusions

We present a case of VZV encephalitis in an immune-compromised middle-aged patient who was primarily diagnosed due to a close temporal association with an onset of dermatomal rashes, which was confirmed with the detection of anti-VZV IgG antibodies in CSF. While PCR can accurately detect viral DNA, it can be negative if a patient is tested later after the onset of symptoms. The most sensitive test is the detection of anti-VZV antibodies in CSF. Therefore, it is highly recommended to perform a lumbar puncture early to rule out encephalitis in patients with altered mental status. Early treatment with IV acyclovir can improve prognosis and facilitate recovery. Since VZV encephalitis is often closely associated with VZV vasculopathy, it is important to consider possible long-term complications, such as stroke, in the long-term follow-up. ESRD is also a significant risk factor for our patient for developing VZV encephalitis and potentially other complications. Hence, it is imperative that clinical practitioners working with these patients should encourage them to get vaccinated for herpes zoster (Shingrix) to avoid life-threatening complications.
